# Evaluation of selected parameters of inflammation, coagulation system, and formation of extracellular neutrophil traps (NETs) in the perioperative period in patients undergoing endovascular treatment of thoracoabdominal aneurysm with a branched device (t-Branch)

**DOI:** 10.3389/fcvm.2023.1153130

**Published:** 2023-09-06

**Authors:** Milena Michalska, Tadeusz Grochowiecki, Aleksandra Wyczałkowska-Tomasik, Leszek Pączek, Tomasz Jakimowicz, Andrzej Cacko, Katarzyna Jama, Albert Stec, Ewa Sikorska, Sławomir Nazarewski, Zbigniew Gałązka

**Affiliations:** ^1^ Department of General, Vascular, Endocrine and Transplant Surgery, Medical University of Warsaw, Warsaw, Poland; ^2^ Department of Immunology, Transplantology, and Internal Diseases, Medical University of Warsaw, Warsaw, Poland; ^3^ Department of Medical Informatics and Telemedicine, Medical University of Warsaw, Warsaw, Poland

**Keywords:** inflammation, extracellular neutrophils traps, neutrophils, thoracoabdominal aortic aneurysm, stent graft placement, t-Branch stent graft

## Abstract

Extracellular Neutrophils Traps (NETs) and their formation, known as NETosis, have become pivotal in the pathogenesis of aortic aneurysm development. This study investigates the NETosis markers with the assessment of selected parameters of inflammation and coagulation system in patients with thoracoabdominal aortic aneurysms in the pre-and postop period undergoing t-Branch stent-graft implantation. The study included 20 patients with thoracoabdominal aortic aneurysms. Three markers double-stranded DNA (dsDNA), single-stranded DNA (ssDNA), and citrullinated H3 histones (Cit-H3) were tested at three-time points from patients’ blood. The parameters of NETosis, inflammation, and coagulation system were examined in the preoperative period (within 24 h before surgery) and in the postoperative period (on the 3rd and 5th postoperative day). Free-circulating DNA (cfDNA) was isolated from the blood using the MagMAXTM Cell-Free DNA Extraction Kit. Double-stranded DNA (dsDNA) and single-stranded DNA (ssDNA) were then quantified using the Qubit dsDNA HS Assay Kit and the Qubit ssDNA Assay Kit. Cit-H3 concentration was determined by enzyme immunoassay ELISA (Cayman). The results revealed the significance of NETs secretion in response to the complex processes after stent-graft implantation. All NET markers increased shortly after surgery, with histones being the first to return to preoperative levels. The lack of normalization of dsDNA and ssDNA levels to preoperative levels by the last postoperative blood collection demonstrates NETs reorganization. The increase in the number of neutrophils was not related to the expansion of postoperative NETosis. The study reveals a new marker of NETosis, ssDNA, that has not been studied so far. The implantation of a stent graft in a patient with TAAA triggers an inflammatory response manifested by an increase in inflammatory parameters. One of the hallmarks of inflammation is the activation of neutrophil extracellular traps.

## 1. Introduction

Based on the anatomic location, aneurysms can develop as a thoracic aortic aneurysm (TAA) which involves descending aorta to the level of the diaphragm, and also as an abdominal aortic aneurysm (AAA) which contains the aorta below renal arteries. Enlargement can occur in visceral arteries part, resulting in a thoracoabdominal aortic aneurysm (TAAA). Multiple variants of aneurysms can be observed from the origin of the left subclavian artery to the aortoiliac bifurcation (1). TAAAs are rare events with an incidence of 5.9 cases per 100,000 persons per year. Patients frequently have significant comorbidities, such as hypertension, coronary heart disease, or chronic obstructive pulmonary disease, which is caused mostly by smoking (2). In both sexes, advanced age is associated with an increased risk, although females acquire TAAAs later in life than men, and females are at an increased danger of rupture (3). Inflammatory cell infiltration into the adventitial wall of patients with aortic aneurysms confirms the participation of processes associated with changes in inflammation parameters (4). The process of aortic aneurysm formation is complex and includes several mechanisms such as medial degeneration of structural proteins (elastin and collagen), which contributes to aortic capacitance and plasticity. Cell infiltration (mainly neutrophils and macrophages) activates a cytokine cascade that activates many proteases. The process of protein degradation coupled with the release of metalloproteinases (MMP) plays a critical role in the inflammatory response in individuals with aneurysms (5). Other processes encompass processes linked with local inflammation and atherosclerotic plaque development (6). It is worth mentioning that inflammatory parameters are raised in patients with aneurysms, which is both a reaction to current processes and may potentially accelerate these processes (7). The creation of an intraluminal thrombus (ILT) is a reflection of the inflammatory response. ILT is a fibrin network that incorporates blood cells, especially neutrophils, platelets, blood proteins, and cellular debris, and is found in varying degrees between the flowing blood and the aorta wall in roughly 75% of all aortic aneurysms (8). The thrombus may operate as a barrier to oxygen transport, causing anoxia of the underlying wall, which may result in reduced thickness and strength, potentially leading to rupture (9). Neutrophil infiltration into the ILT during aortic aneurysm development will thus accumulate neutrophil-derived proteases and result in more severe degradation of the aneurysm (10). Constant interaction between neutrophils and platelet activation within fibrinolysis reflects ILT as a remarkable dynamic *in vivo* process (11), which is the major source of proteolytic activity by neutrophil infiltration (12). Continuous hemostasis/fibrinolysis is essential from the outset of aortic development because plasma proteins such as fibrinogen, d-dimer, and thrombin-antithrombin complex are increased years before clinical manifestations of the disease (13). The accumulation of neutrophils inside the ILT and aortic wall may be linked to the primary pathogenic mechanisms of aneurysmal development which include degradation of the wall, oxidative stress, and inflammatory processes (14). According to the newly revealed capacity of neutrophils to establish extracellular traps, the activity of neutrophils in the pathomechanism of aortic aneurysm growth and thrombus formation is currently under research. Activated neutrophils release neutrophil extracellular traps (NETs) which are a network of extracellular fibers composed of decondensed chromatin with modified histones and neutrophilic granularity (like myeloperoxidase-MPO and neutrophil elastase) which enhance thrombus development by acting as a scaffold that stimulates platelets and coagulation. Neutrophils and NETs play also an indirect role in aortic aneurysm development by influencing other processes which enhance the weakening of the aortic wall. Neutrophils have the ability to affect various mechanisms that lead to aortic enlargements, such as atherosclerosis and inflammation, which are influenced by reactive oxygen species (ROS) and MPO and have an impact on extracellular matrix breakdown by MMP (15). The creation process is known as NETosis. Citrullinated histones and double-stranded DNA (dsDNA) are the most recognizable biomarkers. Previous studies of NETs markers examined cell-free DNA (cfDNA), mostly dsDNA. NETs generation is caused by chromatin releasing beyond the cell containing dsDNA which has the capability to capture additional thrombosis-promoting components such as fibrinogen, fibronectin, von Willebrand factor, factor XII (16), and other morphologic factors including platelets and RBC (17). The ILT decreases and redistributes force in the wall, and the thrombus minimizes pressure in the aneurysmal sac. This reason has been used to promote the usage of endovascular aneurysm repair (EVAR), in which the thrombus is developed inside of the aneurysm and may lead to a reduction in wall compression (12). Endovascular aortic aneurysm repair (EVAR) is a therapy option for individuals with known aortic aneurysms that aims to eliminate the risk of rupture. In recent years, EVAR has been the most commonly used approach for the elective treatment of aortic aneurysms than open repair (18). Branched EVAR (bEVAR) fot TAAA alters the flow in the aorta, while it is a less invasive procedure than the open method, it involves some pathophysiological alterations that may impact the formation of complications. The study's goal is to evaluate during the perioperative phase of bEVAR inflammation and coagulation system conditions in relation to the NETs formation in patients with TAAA.

## 2. Methods

### 2.1. Study design

This is a prospective, single-center observational cohort study supervised by a high-volume center that accomplishes over 150 bEVAR in TAAA annually. Inclusion and exclusion criteria are categorized in Table 1. However, we limited the study group to t-Branch device for better homogeneity. The study design was prepared in accordance with the Helsinki Declaration and was approved by the Ethics Committee of the Medical University of Warsaw (approval number KB/168/2020).

**Table 1:** Represents inclusion and exclusion criteria for patients in this study.

Inclusion criteria	Exclusion criteria
• Patients diagnosed with TAAA • Indications for endovascular aortic repair by t-Branch device • >18 years old • Written informed consent to participate in the study • Platelet count 100 000–450 000/µl	• Coexisting present severe neoplastic disease • Preoperative therapy with oral anticoagulants (dabigatran, rivaroxaban, apixaban, edoxaban) • Coexisting viral or bacterial infection • Patients with other diseases causing advanced organ failure: respiratory/circulatory/kidney/liver • Pregnant women • Patients with poor general condition (> 3 according to ECOG/WHO) • If the treatment of an aortic aneurysm is changed (i.e open repair)

### 2.2. Procedure

All patients underwent successful endovascular repair by using Zenith t-Branch stent graft (Cook Medical, Bloomington, Indiana, USA) with a quadruple vessel framework (divisions for celiac trunk, superior mesenteric artery, and both renal arteries) with proximal and distal components sufficient for aneurysm anatomy. The surgeries were performed after written informed consent from each patient between May 2021 to August 2021 under general anesthesia. Procedures were performed by one high-qualified operator (TJ) in a hybrid operating room via left axillary and femoral access. Indication for stent graft placement was the maximum aortic diameter of ≥55 mm or rapid aneurysm expansion (≥ 10 mm during 12 months).The longitudinal incision was used to dissect the left axillary artery and femoral arteries were exposed from oblique or longitudinal cuts in the groins. The through-and-through wire was placed between the left armpit and right groin. After angiography, adequate endografts were placed including t-Branch in visceral region. After femoral arteries closure adequate bridging stents were inserted into the celiac trunk, superior mesenteric artery, and both major renal arteries via the axillary approach. Coils were used to embolize the accessory renal arteries if needed. Control angiography was performed to check blood flow to the lower limbs, internal iliac arteries, stent graft branches, and visceral arteries. The following bridging covered stents were used: BeGraft (Bentley, Innomed GmbH, Germany) and Fluency (Bard Peripheral Vascular, Tempe, Arizona, USA). Relining of the bridging stents was performed if required by Zilver (Cook Medical Bloomington, Indiana, USA).

### 2.3. Data collection

Data was gathered and then retroactively and anonymously entered into a uniform computer database. Information includes comorbidities and their therapies, demographic data, procedure details, and blood tests which were unified (within 24 h before the operation, on the third and fifth postoperative day).

### 2.4. Perioperative pharmacotherapy

Preoperatively patients underwent antibiotic prophylactic receiving 1 g of cefazolin iv 30–60 min before EVAR. All patients were treated intraoperatively by unfractionated heparin (UFH) infusions based on active clotting time (ACT) values with a target of 200–250 s. Postoperatively, all patients received UFH infusion at the dose determined by activated partial thromboplastin time (APTT) assessment (2–2.5 × normal level) for two days followed by LMWH. DAPT (clopidogrel 75 mg and ASA 75 mg once daily) has been started on the second postoperative day and continued after discharge from the hospital.

### 2.5. Blood collection

Peripheral venous blood samples were taken at three different points: 24 h before EVAR, on a third postoperative day, and on the fifth postoperative day which was commonly the day of discharge from the hospital. In the 24 h preoperative stage and on the fifth postoperative day blood was drawn by aseptic venipuncture from an antecubital vein. On the third postoperative day, blood was drawn from a central puncture implanted in the external jugular vein after EVAR. To eliminate procedural deviations, all blood samples were collected by qualified nurses using a mild tourniquet that was quickly removed, and the samples were well-mixed by lightly inverting the tubes. Four tubes of blood were drawn for plasma (Sarstedt Monovette EDTA ke 2.6 ml) and one tube (Sarstedt Monovette Serum Z 4.9 ml) for serum Table 2 contains the sequence of the blood collection.

**Table 2:** Contains the sequence of the blood collection.

Preoperative phase	−24 h	I-blood collection
Postoperative phase	Third postoperative day	II-blood collection
	Fifth postoperative day	III-blood collection

### 2.6. Plasma for infectious investigation

1 tube of 2.6 ml of blood on EDTA was centrifuged (FrontierTM, Ohaus, FC5718R, Germany) for 20 min at 340 xg at room temperature, then the supernatant was collected with a pasteur pipette (Sarstedt, 86.1171.010) and pipetted 0.5 ml into 2 sterile tubes (Sarstedt, 72.694.006).

### 2.7. Plasma for cf, dsDNA and cf, ssDNA investigation

3 tubes of 2.6 ml of EDTA blood were centrifuged for 10 min at 1,600 xg at 4°C, then the supernatant was gathered with a pasteur pipette (Sarstedt, 86.1171.010) and placed in a tube (Sarstedt, 62.558.201) and centrifuged again for 10 min at 16,000 xg at 4°C, then the supernatant was harvested up with a pasteur pipette (Sarstedt, 86.1171.010) and pipetted 2.0 ml into 2 sterile tubes (Sarstedt, 72.694.006).

### 2.8. Serum for Cit-H3 investigation

Blood obtained on the clot was intended to allow at room temperature for at least 30 min before centrifugation at 85 xg for 10 min at room temperature, and the supernatant was collected using a pasteur pipette (Sarstedt, 86.1171.010) and pipetted 0.5 ml to two sterile tubes (Sarstedt, 72.694.006). The material was obtained and stored in a freezer (Eppendorf Cryocube F740hi) at −80°C until analysis.

### 2.9. Quantitative assessment of free circulating double-stranded DNA (cf, dsDNA) and single-stranded DNA (cf, ssDNA)

The first step was the isolation of free circulating DNA (cfDNA) made with the MagMAXTM Cell-Free DNA Isolation Kit from Applied Biosystems (Cat. No. A29319, Applied Biosystems). The material for the research was blood plasma. 4 ml of edetate plasma was used for isolation. Isolation was carried out following the manufacturer's instructions. The next step was the quantification of double-stranded DNA (dsDNA) and single-stranded DNA (ssDNA). In a sample of the isolated cfDNA, the concentration of double-stranded DNA was measured. This was performed using the Qubit dsDNA HS Assay Kit (Cat. No. Q32851, Invitrogen) and the Qubit ssDNA Assay Kit from Invitrogen (Cat. No. Q10212, Invitrogen). The study was conducted in doublets. The final measurement was made on Qubit 4 Fluorometer from Invitrogen.

### 2.10. Determining the concentration of Cit-H3

The Cit-H3 concentrations were determined using an enzyme immunoassay using the ELISA method. Cayman's Citrullinated Histone H3 (Clone 11D3) (Cat. No. 501620, Cayman) ELISA kit was used in the investigation. Serum served as the research's initial stage. The test was performed in doubles. The final measurement was placed at a wavelength of *λ* = 450 nm on a BioTek Power Wave XS spectrophotometer by Bio-Tek Instruments. Calculation of the Cit-H3 concentration in the test tubes was performed with the KCjuniorWin computer program, using the standard curve method.

### 2.11. Determining the ratio of NETs marker to quantity of neutrophils

In order to investigate whether the intensity of NETs secretion depends on the number of neutrophils, we introduced the ratio of marker concentration (dsDNA, ssDNA, Cit-H3) per 1,000 neutrophils. These ratios between blood collections were compared.

### 2.12. Statistical analysis

Data were analyzed using commercial software (Statsoft Statistica 13.3; Tibco Software, Palo Alto, CA, USA). Descriptive statistics are given for all data. The normality assumption was examined using Shapiro–Wilk tests. The distributions of most sample parameters were not normal, and Levene tests showed heteroscedasticity in these cases. Therefore, nonparametric methods were used for all samples like Spearman's rank correlation. Wilcoxon signed-rank tests were used to compare values of analyzed parameters in samples from patients. Significance was defined at *p* < 0.05.

### 2.13. Material

In 20 patients (85% male, 15% female), the endovascular aortic repair was performed. The average age of qualified patients was 70.65 ± 6.23 years, of which 50% were overweight or obese. In 35% of patients, pure obesity was observed. The majority of patients, up to 60%, had smoked cigarettes in the past, and the average time without smoking was 9.3 ± 7.57 years. Currently, 35% of patients smoke. The average pack-year period of smokers is 28.98 ± 17.65 years. Only one patient was not hooked on smoking. Table 3 summarizes the study's enrolled patients' characteristics. The most frequently reported co-morbidities are hypertension (85%) and cardiological diseases (65%), of which only 25% had myocardial infarction). All patients diagnosed with hypertension (85%) were on antihypertensive therapy. Patients frequently used beta-blockers (65%), calcium antagonists (40%), and diuretics (40%). ACEI (35%) and ARB (25%) were less widely used medications. Hyperlipidemia was diagnosed in 65% of patients and treated in 60%. Among the medications, 55% were statins and 10% were fibrates. None of the patients was diagnosed with diabetes. Other comorbidities and their frequency are presented in Table 4. Regarding drugs affecting coagulation, 50% of patients received ASA, 10% oral inhibitors of platelet P2Y12, and 5% vitamin K antagonists. Table 5 depicts the patients' preoperative medication treatment. Symptoms associated with aneurysm patients were reported in 80%, of which the most common complaints were abdominal pain (40%) and back pain (35%). The aneurysm was felt by 35% of the individuals. Table 6 displays symptoms reported by participants. The overall duration of hospitalization was 13.9 ± 6.92 days, with the postoperative phase lasting 9 ± 7.36 days on average. Before surgery, all patients received a PCR test for COVID, which was 100% negative. 65% of the participants in the research had already undergone surgery in their lifetime, and 30% had secondary aneurysm treatment. Table 7 outlines the characteristics of the study's recruited participants. Crawford's categorization identified type I in 5% of patients, type II in 0%, type III in 10%, and type IV in 85% of participants. All patients were operated on under general anesthesia, and all were given prophylactic cefazolin before to surgery. In no case, postoperative wound infection was found. Six patients (30%) had significant stenosis of any aortic branch (including twice stenosis of the celiac artery, twice stenosis of the internal iliac artery, stenosis of the left renal artery, stenosis of the right renal artery) 15% had additional renal arteries, and 10% underwent embolization. The average amount of blood lost was 205 ± 99.87 ml. The operation took an average of 137.5 ± 19.1 min. Table 8 illustrates the operation details and characteristics of TAAA.

**Table 3:** Summarizes the study's enrolled patients’ characteristics.

Variable	Mean ± SD; Median (IQR) or No (%)
Age [years]	70.65 ± 6.23; 71.50 (65.50–75.00)
BMI [kg/m^2^]	26.82 ± 4.68; 24.89 (23.18–30.95)
BMI >25	10 (50%)
BMI >30	7 (35%)
Sex	
Female	3 (15%)
Male	17 (85%)
Smoker status	
Never	1 (5%)
Past	12 (60%)
Time without smoking (years)	9.3 ± 7.57; 5.00 (5–20)
Current	7 (35%)
Pack-year [years]	28.98 ± 17.65; 27.00 (17.50–40)

**Table 4:** Summarizes the morbidity of research participants.

Comorbidities	No (%)
Cardiological diseases	13 (65%)
Including only myocardial infarction	5 (25%)
Hypertensive	17 (85%)
Including only hypertensive therapy	17 (85%)
Hyperlipidemia	13 (65%)
Including only lipid-lower therapy	12 (60%)
Pulmonary diseases	7 (35%)
Including only COPD	3 (15%)
Neurological diseases	3 (15%)
Including only stroke	2 (10%)

**Table 5:** Depicts the patients’ preoperative medication treatment.

Drugs	No (%)
Statins	11 (55%)
Fibrates	2 (10%)
β*-blockers*	13 (65%)
ACEI (angiotensin-converting-enzyme inhibitors)	7 (35%)
ARB (angiotensin II receptor blockers)	5 (25%)
Calcium antagonist	8 (40%)
Diuretics	8 (40%)
ASA (acetylsalicylic acid)	10 (50%)
Anti-PLT P2Y12 (oral inhibitors of platelets P2Y12)	2 (10%)
Vit K antagonist	1 (5%)
NOAC (non-vitamin K antagonist oral anticoagulants)	0 (0%)
Antidiabetic	1 (5%)
PPI (Proton-pump inhibitor)	3 (15%)
Anti-prostatic hyperplasia drugs	4/17 (23.53%)

**Table 6:** Displays symptoms reported by participants.

Symptoms	No (%)
Signs and symptoms	16 (80%)
Back pain (lumbar pain)	7 (35%)
Abdominal pain	8 (40%)
Groin pain	2 (10%)
Constipation	4 (20%)
Cough	1 (5%)
Palpable aneurysm by patients	7 (35%)

**Table 7:** Outlines the characteristics of the study's recruited participants.

Variable	Mean ± SD; Median (IQR) or No (%)
AA family history	3 (15%)
Second AA	6 (30%)
Operations	13 (65%)
Negative SARS-CoV-2 preoperative PCR	20 (100%)
Days of hospitalization after the surgery [days]	9.0 ± 7.36; 7.00 (5–9)

**Table 8:** Illustrates the operation details and characteristics of TAAA.

Variable	Mean ± SD; Median (IQR) or No (%)
Type (I-IV)	
I	1/20 (5%)
II	0/20 (0%)
III	2/20 (10%)
IV	17/20 (85%)
Accessory renal arteries	3 (15%)
Embolization of accessory renal	2 (10%)
Estimated blood loss [ml]	205 ± 99.87; 175.00 (150–250)
Procedure time [min]	137.5 ± 19.1; 135.00 (120–150)
Fluoroscopy time [min]	27.4 ± 11.9; 24.30 (19–34.25)
Radiation dose [mGy]	873.88 ± 543.49; 678.30 (508.1–1110.65)
Radiation dose [μGy·cm^2^]	8203.81 ± 4086.13; 7189.250 (5476.1–10295.4)
Critical stenosis of any branch of the aorta	6 (30%)
General anesthesia	20 (100%)
Antibiotic prevention (cefazolin)	20 (100%)
Number of drains	
2	12 (60%)
3	8 (40%)

## 3. Results

**Figure 1:**
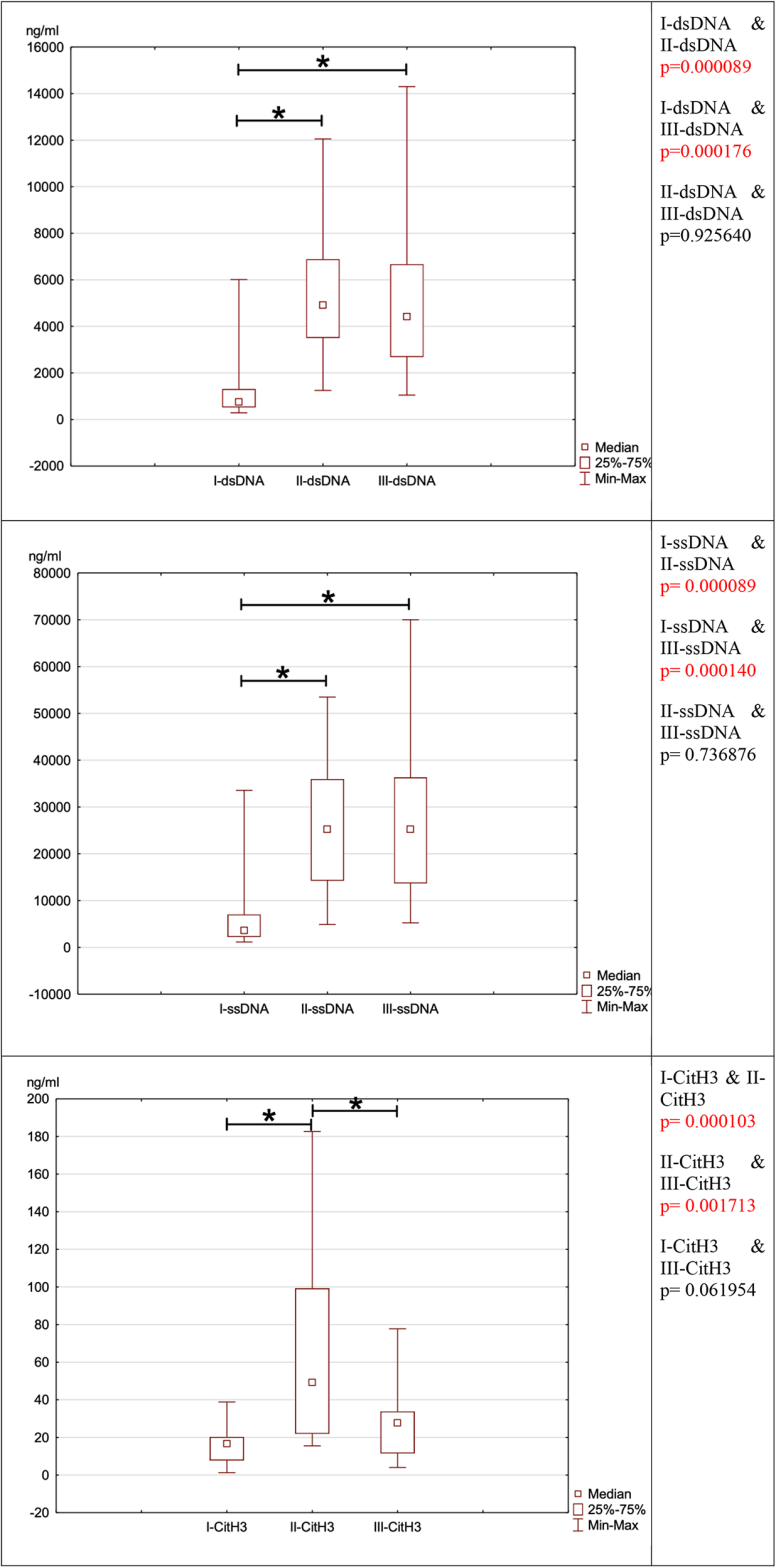
Depicts the evolution of neutrophil network markers over time. (**A**)- level of neutrophil network markers such as dsDNA, ssDNA and Cit-H3 presented over time. (**B**)- the ratio between ssDNA/dsDNA between blood draws. (**C**)- a difference in concentration between dsDNA and ssDNA in individual blood samples I, II, and III.

**Figure 2:**
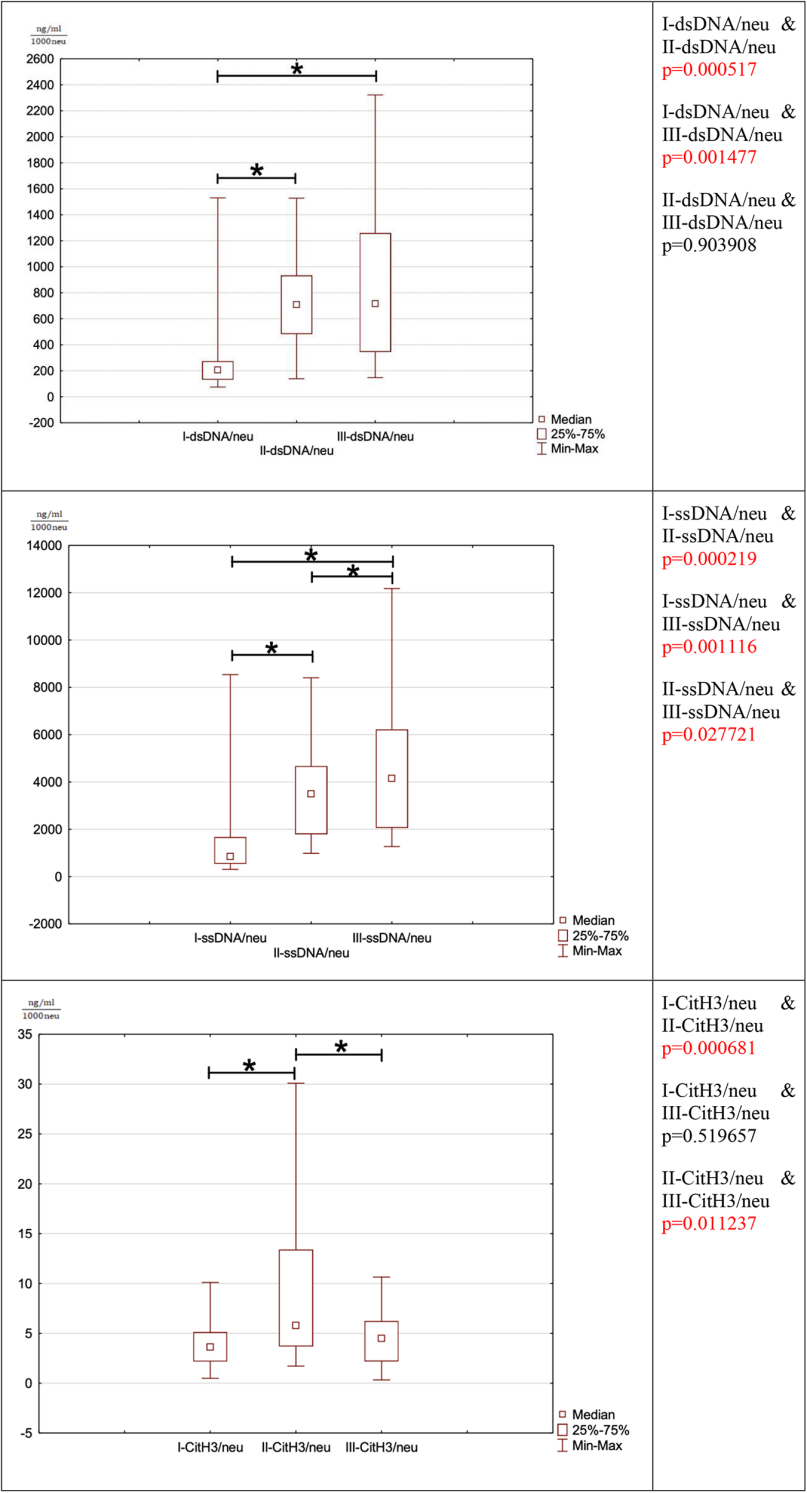
Iillustrates the evolution of NET marker ratio changes per 1,000 neutrophils.

NETosis marker was shown in Figure 1. NETosis markers per 1,000 neutrophils were shown in Figure 2.

**Figure:**
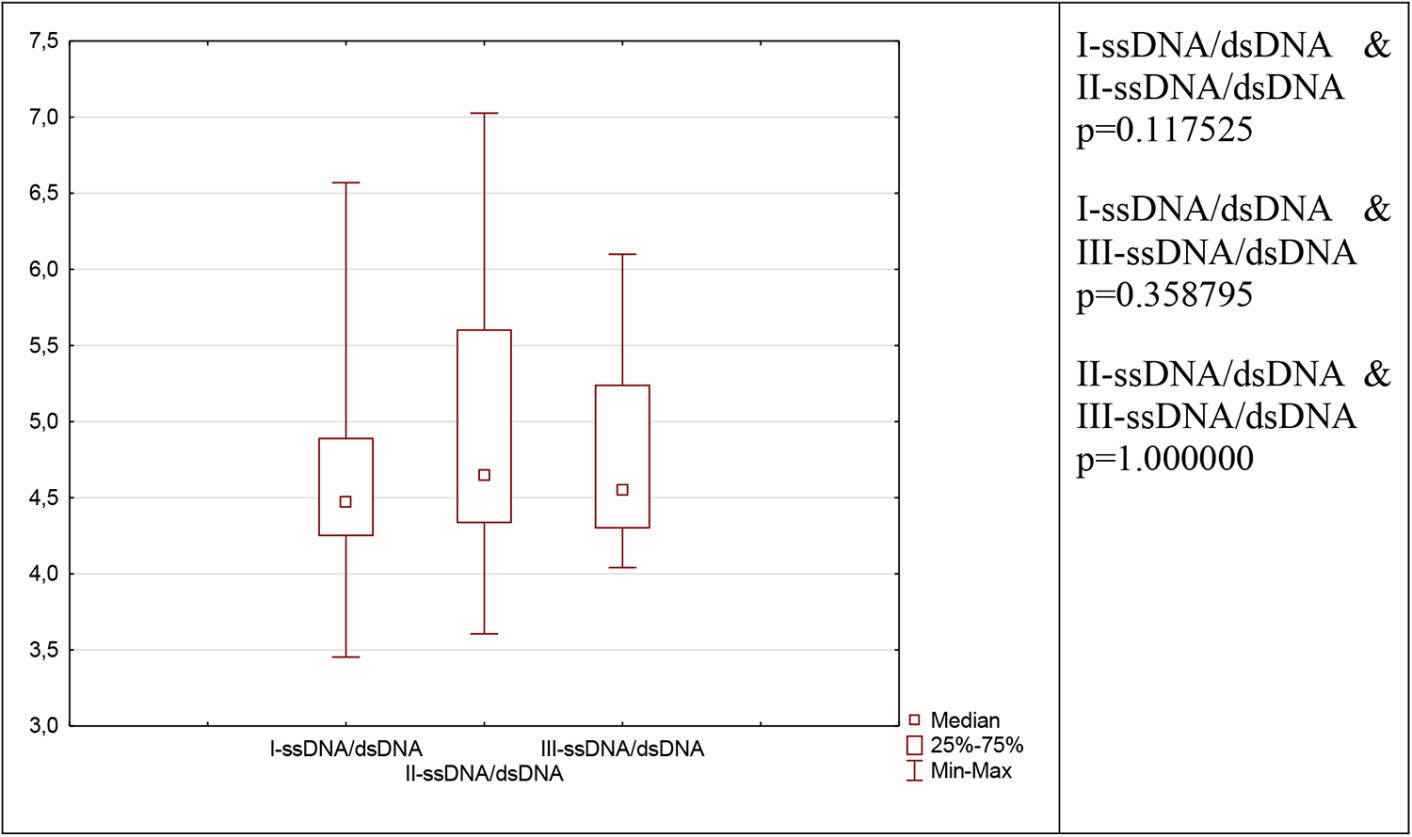


**Figure:**
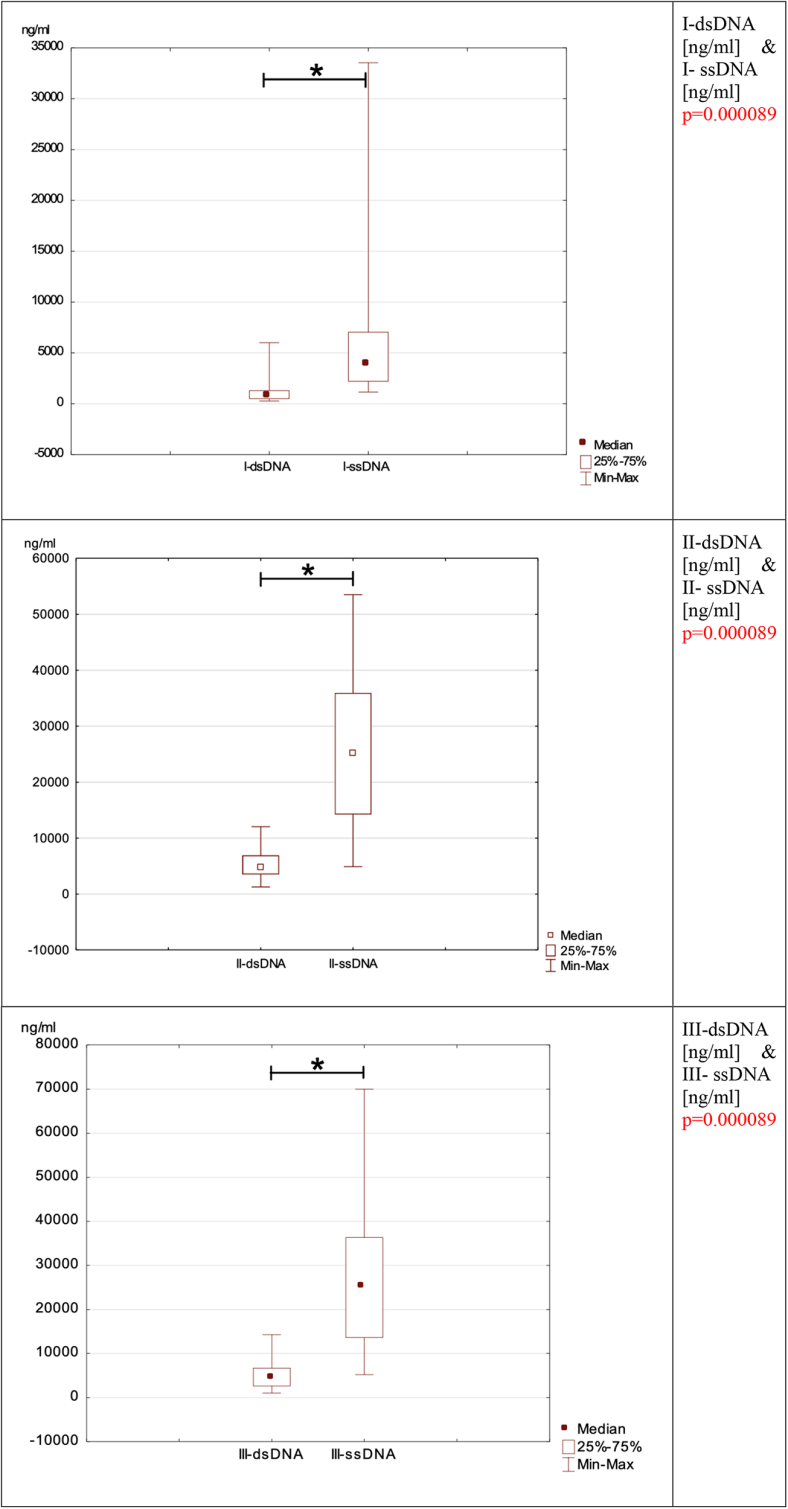


### 3.1. dsDNA

In the preoperative period, the median concentration of dsDNA is 757.75 (IQR: 520.75–1310.00) The concentration of dsDNA on postoperative days were significantly higher than in the preoperative period. (III day: dsDNA: median: 4907.50; IQR: 3500.00–6892.50; I-dsDNA & II-dsDNA *p* = 0.000089, V day: dsDNA: median: 4420.00; IQR:2682.50–6677.50; I-dsDNA & III-dsDNA *p* = 0.000176) This marker's concentration does not return to preoperative levels until the fifth postoperative day (II-dsDNA & III-dsDNA *p* = 0.925640).

### 3.2. ssDNA

Because the preoperative ssDNA concentration was the lowest (ssDNA: 3625.00; IQR:2212.50–7057.50) compared to the postoperative period, the second marker-ssDNA responded similarly to dsDNA. In the postoperative period, the concentration of ssDNA increased significantly. In the postoperative period, the concentration of ssDNA increased (III day: median: 25250.00; IQR:14225.00–35975.00; I-ssDNA & II-ssDNA *p* = 0.000089, V day: median: 25225.00; IQR: 13650.00–36325.00; I-ssDNA & III-ssDNA *p* = 0.000140) Likewise, to dsDNA, the concentration of ssDNA did not decrease over the preoperative period (II-ssDNA & III-ssDNA *p* = 0.736876).

### 3.3. Cit-H3

Cit-H3 reacted differently from the other two markers. Cit-H3 concentration increased statically on the third postoperative day compared to the preoperative period (preoperative Cit-H3: median: 16.67; IQR:7.76–20.30; III day: median: 49.23; IQR:21.87–99.27; I-Cit-H3 & II-Cit-H3 *p* = 0.000103). The postoperative concentration of Cit-H3 returned to preoperative concentration on the fifth postoperative day (V day: median: 27.73; IQR:11.44–33.92; II-CitH3 & III-CitH3 *p* = 0.001713, I-Cit-H3 & III-Cit-H3 *p* = 0.061954).

### 3.4. ssDNA and dsDNA

When comparing the concentrations of ssDNA and dsDNA in each blood sample, the ssDNA concentration is significantly higher than the dsDNA concentration (I-dsDNA [ng/ml] & I-ssDNA [ng/ml] *p* = 0.000089, II-dsDNA [ng/ml] & II-ssDNA [ng/ml] *p* = 0.000089, III-dsDNA [ng/ml] & III-ssDNA [ng/ml] *p* = 0.000089).

### 3.5. ssDNA/dsDNA

The ratio of ssDNA to dsDNA reveals an intriguing relationship since it remains constant level in each collection (I-ssDNA/dsDNA & II-ssDNA/dsDNA *p* = 0.117525; I-ssDNA/dsDNA & III-ssDNA/dsDNA *p* = 0.358795; II-ssDNA/dsDNA & III-ssDNA/dsDNA *p* = 1). Despite the postoperative elevation in ssDNA and dsDNA, this ratio remains unchanged and is relatively comparable in each patient (I-ssDNA/dsDNA IQR = 4.27–4.86; II- ssDNA/dsDNA IQR = 4.33–5.50, III-ssDNA/dsDNA IQR = 4.37–5.21).

A very strong and significant association between ssDNA and dsDNA is seen according to the correlation measurements. It was *r* = 0.975555 (*p* = 0.000000) throughout the preoperative period, *r* = 0.908271 (*p* = 0.000000) on the third postoperative day, and *r* = 0.863158 (*p* = 0.000001) on the fifth postoperative day. Table 9 displays the Spearmen correlations between ssDNA and dsDNA.

**Table 9:** Displays the spearman correlations between ssDNA and dsDNA. The correlations shown have statistical significance <0.05. ns stands for not significant.

	I-dsDNA [ng/ml]	II-dsDNA [ng/ml]	III-dsDNA [ng/ml]
I-ssDNA [ng/ml]	0.9,75,555	ns	ns
II-ssDNA [ng/ml]	ns	0.9,08,271	0.5,48,872
III-ssDNA [ng/ml]	ns	0.6,75,188	0.8,63,158

### 3.6. dsDNA/neu

The ratio of dsDNA to 1,000 neutrophils is significantly increased between the preoperational phase to the third and fifth postoperative period (I-dsDNA/neu & II-dsDNA/neu *p* = 0.000517 (I-dsDNA/neu & III-dsDNA/neu *p* = 0.001477). The ratio does not return to the preoperative range until the fifth postoperative day. The postoperative phase, there is no significant difference in this ratio between the third and fifth postoperative day (II-dsDNA/neu & III-dsDNA/neu *p* = 0.903908).

### 3.7. dsDNA/neu

The ratio of ssDNA to 1,000 neutrophils responds similarly to dsDNA since it is significantly increased between the preoperative the postoperative period (I-ssDNA/neu & II-ssDNA/neu *p* = 0.000219, I-ssDNA/neu & III-ssDNA/neu *p* = 0.001116). There was an increase in the ratio between the third and fifth postoperative day (II-ssDNA/neu & III-ssDNA/neu *p* = 0.027721). The ratio does not return to the preoperative range until the fifth postoperative day.

### 3.8. Cit-H3/neu

The ratio of the number of Cit-H3 per 1,000 neutrophils increased statistically between the preoperative period and the third postoperative day (I-CitH3/neu & II-CitH3/neu *p* = 0.000681). In the postoperative period, a significant decrease in the ratio between the third and fifth postoperative days was observed (II-CitH3/neu & III-CitH3/neu *p* = 0.011237). This ratio was normalized to the preoperative state in the fifth postoperative period (I-CitH3/neu & III-CitH3/neu *p* = 0.519657).

### 3.9. Laboratory findings

Table 10 shows the results of patients’ laboratory testing.

**Table 10:** Shows the results of patients’ laboratory testing. ns stands for not significant.

Parameters	Preoperative (−24 h) Mean ( ± SD) Median IQR	Postoperative (III Day) Mean ( ± SD) Median IQR	Postoperative (V Day) Mean ( ± SD) Median IQR	*p*-value
WBC [10^3^/µl]	7.73 ± 2.11	10.70 ± 3.56	10.03 ± 3.74	I-WBC [10^3^/µl] & II-WBC [10^3^/µl] *p* = 0.001713
	7.26	10.73	10.33	I-WBC [10^3^/µl] & III-WBC [10^3^/µl] *p* = 0.002495
	6.23–9.10	7.77–13.37	6.96–12.76	II-WBC [10^3^/µl] & III-WBC [10^3^/µl] *p* = ns
NEU [10^3^/µl]	4.31 ± 1.63	7.84 ± 3.06	6.91 ± 6.91	I-NEU [10^3^/µl] & II-NEU [10^3^/µl] *p* = 0.0,00,455
	4.27	7.23	6.51	I-NEU [10^3^/µl] & III-NEU [10^3^/µl] *p* = 0.004970
	3.05–4.91	5.68–9.74	5.00–7/90	II-NEU [10^3^/µl] & III-NEU [10^3^/µl] *p* = ns
LYM [*10^3^*/µl]	2.48 ± 1.42	1.64 ± 1.64	1.98 ± 1.42	I-LYM [10^3^/µl] & II-LYM [10^3^/µl] *p* = 0.000625
	2.32	1.27	1.60	I-LYM [10^3^/µl] & III-LYM [10^3^/µl] *p* = 0.004970
	1.55–2.65	0.93–2.00	1.39–2.30	II-LYM [10^3^/µl] & III-LYM [10^3^/µl] *p* = 0.031105
neu/limf ratio	2.15 ± 1.49	6.89 ± 4.94	4.92 ± 5.14	I-NLR & II-NLR *p* = 0.000463
	1.87	5.62	3.41	I-NLR & III-NLR *p* = 0.009563
	1.20–2.35	3.59–9.68	2.54–5.49	II-NLR & III-NLR *p* = 0.007399
MON [*10^3^*/µl]	1.11 ± 1.48	1.37 ± 1.49	1.44 ± 1.48	I-MON [10^3^/µl] & II-MON [10^3^/µl] *p* = 0.007399
	0.72	1.11	1.00	I-MON [10^3^/µl] & III-MON [10^3^/µl] *p* = 0.000581
	0.60–1.12	0.62–1.52	0.90–1.60	II-MON [10^3^/µl] & III-MON [10^3^/µl] *p* = ns
EOS [*10^3^*/µl]	0.62 ± 1.59	0.50 ± 1.61	0.62 ± 1.63	I-EOS [10^3^/µl] & II-EOS [10^3^/µl] *p* = 0.006211
	0.21	0.12	0.17	I-EOS [10^3^/µl] & III-EOS [10^3^/µl] *p* = ns
	0.16–0.42	0.04–0.24	0.10–0.50	II-EOS [10^3^/µl] & III-EOS [10^3^/µl] *p* = 0.007745
BASO [*10^3^*/µl]	0.39 ± 1.63	0.38 ± 1.63	0.40 ± 1.67	I-BASO [10^3^/µl] & II-BASO [10^3^/µl] *p* = 0.014736
	0.03	0.02	0.01	I-BASO [10^3^/µl] & III-BASO [10^3^/µl] *p* = 0.02096
	0.01–0.05	0.00–0.03	0.00–0.02	II-BASO [10^3^/µl] & III-BASO [10^3^/µl] *p* = ns
NEU [%]	52.35 ± 15.65	73.91 ± 8.60	66.76 ± 10.38	I-NEU [%] & II-NEU [%] *p* = 0.000189
	52.95	73.85	62.90	I-NEU [%] & III-NEU [%] *p* = 0.003307
	47.15–59.10	67.75–79.85	60.70–72.70	II-NEU [%] & III-NEU [%] *p* = 0.001370
LYM [%]	32.58 ± 13.61	16.73 ± 14.61	21.37 ± 14.46	I-LYM [%] & II-LYM [%] *p* = 0.000214
	29.55	13.05	18.30	I-LYM [%] & III-LYM [%] *p* = 0.001009
	24.50–38.85	8.35–19.00	13.70–24.50	II-LYM [%] & III-LYM [%] *p* = 0.005317
MON [%]	13.55 ± 14.10	13.05 ± 14.41	14.68 ± 14.42	I-MON [%] & II-MON [%] *p* = ns
	10.50	10.45	12.10	I-MON [%] & III-MON [%] *p* = ns
	9.10–12.30	8.50–12.90	9.80–13.50	II-MON [%] & III-MON [%] *p* = ns
EOS [%]	7.01 ± 15.63	5.11 ± 15.96	6.29 ± 16.19	I-EOS [%] & II-EOS [%] *p* = 0.000900
	3.25	1.40	2.20	I-EOS [%] & III-EOS [%] *p* = 0.107509
	2.25–5.15	0.40–2.80	1.30–3.90	II-EOS [%] & III-EOS [%] *p* = 0.003090
BASO [%]	4.14 ± 16.14	3.84 ± 16.21	4.05 ± 16.62	I-BASO [%] & II-BASO [%] *p* = 0.000581
	0.50	0.20	0.30	I-BASO [%] & III-BASO [%] *p* = 0.006134
	0.40–0.65	0.10–0.30	0.00–0.40	II-BASO [%] & III-BASO [%] *p* = ns
RBC [*10^6^*/µl]	7.70 ± 15.31	7.06 ± 15.46	6.89 ± 15.50	I-RBC [10^6^/µl] & II-RBC [10^6^/µl] *p* = 0.000155
	4.36	3.44	3.36	I-RBC [10^6^/µl] & III-RBC [10^6^/µl] *p* = 0.000132
	3.77–4.57	3.13–4.18	2.99–4.10	II-RBC [10^6^/µl] & III-RBC [10^6^/µl] *p* = 0.029
HGB [g/dl]	16.25 ± 13.43	14.28 ± 13.89	13.82 ± 14.00	I-HGB [g/dl] & II-HGB [g/dl] *p* = 0.000155
	13.30	10.65	10.20	I-HGB [g/dl] & III-HGB [g/dl] *p* = 0.000132
	11.90–14.40	9.65–12.60	9.25–12.90	II-HGB [g/dl] & III-HGB [g/dl] *p* = ns
HCT [%]	40.80 ± 9.34	34.39 ± 10.58	33.19 ± 11.01	I-HCT [%] & II-HCT [%] *p* = 0.000132
	39.55	30.70	29.40	I-HCT [%] & III-HCT [%] *p* = 0.000132
	35.20–42.50	28.10–37.00	26.95–37.25	II-HCT [%] & III-HCT [%] *p* = ns
MCV [fl]	90.66 ± 6.78	88.92 ± 6.07	89.88 ± 6.25	I-MCV [fl] & II-MCV [fl] *p* = 0.005492
	91.90	89.65	90.75	I-MCV [fl] & III-MCV [fl] *p* = ns
	88.40–93.55	85.50–92.50	86.60–93.00	II-MCV [fl] & III-MCV [fl] *p* = ns
MCH [pg]	33.16 ± 9.53	33.09 ± 9.50	33.37 ± 9.43	I-MCH [pg] & II-MCH [pg] *p* = ns
	31.05	31.10	31.40	I-MCH [pg] & III-MCH [pg] *p* = ns
	29.85–32.65	30.05–32.20	30.10–32.50	II-MCH [pg] & III-MCH [pg] *p* = 0.011286
MCHC [g/dl]	35.01 ± 9.68	35.38 ± 9.89	35.31 ± 9.85	I-MCHC [g/dl] & II-MCHC [g/dl] *p* = ns
	34.00	34.40	34.350	I-MCHC [g/dl] & III-MCHC [g/dl] *p* = ns
	33.30–34.50	33.95–34.75	34.05–34.80	II-MCHC [g/dl] & III-MCHC [g/dl] *p* = ns
RDW-CV [%]	17.25 ± 13.13	17.47 ± 13.07	17.47 ± 13.07	I-RDW-CV [%] & II-RDW-CV [%] *p* = ns
	13.90	14.40	15.15	I-RDW-CV [%] & III-RDW-CV [%] *p* = ns
	13.10–15.60	13.10–15.60	13.20–15.95	II-RDW-CV [%] & III-RDW-CV [%] *p* = ns
PLT [10*^3^*/µl]	211.49 ± 66.51	146.84 ± 60.21	152.59 ± 68.64	I-PLT [10^3^/µl] & II-PLT [10^3^/µl] *p* = 0.000132
	203.00	129.00	136.50	I-PLT [10^3^/µl] & III-PLT [10^3^/µl] *p* = 0.000132
	179.50–242.00	114.00–166.00	116.00–172.00	II-PLT [10^3^/µl] & III-PLT [10^3^/µl] *p* = ns
MPV [fl]	13.56 ± 13.97	13.57 ± 13.97	13.70 ± 13.94	I-MPV [fl] & II-MPV [fl] *p* = ns
	10.80	10.35	10.20	I-MPV [fl] & III-MPV [fl] *p* = ns
	10.15–11.05	9.55–11.60	9.70–11.50	II-MPV [fl] & III-MPV [fl] *p* = ns
PCT [%]	0.23 ± 0.06	0.15 ± 0.05	0.16 ± 0.07	I-PCT [%] & II-PCT [%] *p* = 0.000196
	0.22	0.15	0.15	I-PCT [%] & III-PCT [%] *p* = 0.000151
	0.20–0.25	0.12–0.16	0.12–0.017	II-PCT [%] & III-PCT [%] *p* = ns
PDW [fl]	13.36 ± 2.55	14.04 ± 2.43	15.01 ± 3.60	I-PDW [fl] & II-PDW [fl] *p* = ns
	12.55	14.30	15.10	I-PDW [fl] & III-PDW [fl] *p* = 0.013042
	12.25–13.50	12.70–15.20	12.10–17.35	II-PDW [fl] & III-PDW [fl] *p* = ns
CRP [mg/L]	21.86 ± 31.19	143.58 ± 54.35	141.28 ± 52.07	I-CRP [mg/L] & II-CRP [mg/L] *p* = 0.000140
	7.50	131.2	152.50	I-CRP [mg/L] & III-CRP [mg/L] *p* = 0.000120
	2.75–35.10	112.10–180.70	104.30–178.90	II-CRP [mg/L] & III-CRP [mg/L] *p* = ns
Prothrombin time [s]	12.40 ± 1.34	13.79 ± 1.48	12.67 ± 1.06	I-PROTHROMBIN TIME [s] & II- PROTHROMBIN TIME [s] *p* = 0.000536
	12.10	13.70	12.30	I- PROTHROMBIN TIME [s] & III- PROTHROMBIN TIME [s] *p* = ns
	1.70–12.55	12.30–15.20	11.70–12.55	II- PROTHROMBIN TIME [s] & III- PROTHROMBIN TIME [s] *p* = 0.000438
Prothrombin index [%]	96.95 ± 9.12	87.21 ± 9.52	90.25 ± 20.19	I-PROTHROMBIN INDEX [%] & II- PROTHROMBIN INDEX [%] *p* = 0.000536
	98.00	87.00	97.00	I- PROTHROMBIN INDEX [%] & III- PROTHROMBIN INDEX [%] *p* = ns
	95.00–102.00	78.00–97.00	87.00–102.00	II- PROTHROMBIN INDEX [%] & III- PROTHROMBIN INDEX [%] *p* = 0.000438
INR	1.04 ± 0.12	1.17 ± 0.12	1.07 ± 0.09	I-INR & II-INR *p* = 0.000342
	1.02	1.15	1.03	I-INR & III-INR *p* = ns
	0.98–1.06	1.03–1.28	0.98–1.11	II-INR & III-INR *p* = 0.000293
Fibrinogen [mg/dl]	406.65 ± 129.78	441.10 ± 88.3	491.11 ± 139.18	I-FIBRINOGEN [mg/dl] & II- FIBRINOGEN [mg/dl] *p* = ns
	390.50	443.00	450.00	I- FIBRINOGEN [mg/dl] & III- FIBRINOGEN [mg/dl] *p* = 0.036386
	298.00–472.00	384.50–490.50	376.00–609.00	II- FIBRINOGEN [mg/dl] & III- FIBRINOGEN [mg/dl] *p* = ns
D-dimer [ng/ml]	4,113.25 ± 2,734.32	6,236.80 ± 2,793.72	5,839.42 ± 2,577.47	I-D-DIMER [ng/ml] & II-D-DIMER [ng/ml] *p* = 0.002225
	3,460.50	6,309.00	5,533.00	I-D-DIMER [ng/ml] & III-D-DIMER [ng/ml] *p* = 0.022232
	2,012.75–5,662.50	3,605.50–8,481.50	3,276.00–7,364.50	II-D-DIMER [ng/ml] & III-D-DIMER [ng/ml] *p* = ns
APTT [s] (activated partial thromboplastin time)	33.31 ± 9.34	43.87 ± 20.98	31.31 ± 4.30	I-APTT [s] & II-APTT [s] *p* = 0.030366
	30.45	32.20	31.60	I-APTT [s] & III-APTT [s] *p* = 0.747500
	28.95–3.55	30.00–59.89	28.50–34.55	II-APTT [s] & III-APTT [s] *p* = 0.006211
APTT RATIO	1.11 ± 0.31	1.46 ± 0.70	1.04 ± 0.14	I-APTT RATIO & II-APTT RATIO *p* = 0.036386
	1.02	1.07	1.05	I-APTT RATIO & III-APTT RATIO *p* = ns
	0.97–1.15	1.00–2.00	0.95–1.16	II-APTT RATIO & III-APTT RATIO *p* = 0.007449
Procalcitonin [ng/ml]	0.05 ± 0.03	1.03 ± 3.51	0.45 ± 1.08	I-PROKALCITONIN [ng/ml] & II- PROKALCITONIN [ng/ml] *p* = 0.000,293
	0.05	0.02	0.14	I- PROKALCITONIN [ng/ml] & III- PROKALCITONIN [ng/ml] *p* = 0.000233
	0.03–0.05	0.14–0.25	0.12–0.24	II- PROKALCITONIN [ng/ml] & III- PROKALCITONIN [ng/ml] *p* = ns

#### 3.9.1. WBC

The concentration of white blood cells (WBC) changes between the period before and after stent administration. The WBC concentration rises statistically after the t-Branch placement (on the third and fifth days, I-WBC [10^3^/µl] & II-WBC [10^3^/µl] *p* = 0.001713.

I-WBC [10^3^/µl] & III-WBC [10^3^/µl] *p* = 0.002495) and does not return to preoperative levels until the fifth postoperative day (II-WBC [10^3^/µl] & III-WBC [10^3^/µl] *p* = 0.304587).

#### 3.9.2. NEU

Neutrophils (NEU), which are the largest fraction of WBC and account for less than 70% of WBC, act similarly to total WBC. The concentration of neutrophils increases in the postoperative phase (I-NEU [10^3^/µl] & II-NEU [10^3^/µl] *p* = 0.000455, I-NEU [10^3^/µl] & III-NEU [10^3^/µl] *p* = 0.004970) and does not decrease until the last sample collection (II-NEU [10^3^/µl] & III-NEU [10^3^/µl] *p* = 0.092861). The percentage of neutrophils reacts slightly differently, the percentages increase statistically between the preoperative period and the third postoperative day (I-NEU [%] & II-NEU [%] *p* = 0.000189) and decreasing significantly between the third and fifth postoperative days II-NEU [%] & III-NEU [%] *p* = 0.001370). Despite this, the percentage of neutrophils is still higher on the fifth postoperative day than it was before surgery (I-NEU [%] & III-NEU [%] *p* = 0.003307).

#### 3.9.3. LYM

Lymphocytes (LYM), the second most abundant WBC fraction after neutrophils, undergo significant changes than neutrophils. In contrast to the preoperative period, lymphocyte concentration drops on the third postoperative day (I-LYM [10^3^/µl] & II-LYM [10^3^/µl] *p* = 0.000625). Following that, leukocytes statistically increased between the third and fifth postoperative days (II-LYM [10^3^/µl] & III-LYM [10^3^/µl] *p* = 0.031105), but do not approach preoperative levels (I-LYM [10^3^/µl] & III-LYM [10^3^/µl] *p* = 0.004970). This indicates that the lowest concentration of leukocytes was found in the obtained samples on the third postoperative day. The proportion of lymphocyte concentrations between the individual in pre-and postoperative days revealed the same relationships. The percentage of lymphocytes decreased significantly after surgery (I-LYM [%] & II-LYM [%] *p* = 0.000214, I-LYM [%] & III-LYM [%] *p* = 0.001009). On the third postoperative day, the concentration was the lowest. Up to the fifth postoperative day, the percentage of lymphocytes did not increase to pre-operation levels despite a significant increase between the third and fifth post-operation days (II-LYM [%] & III-LYM [%] *p* = 0.005317).

#### 3.9.4. NLR

The mutual ratio of neutrophils and lymphocytes, known as the NLR, fluctuates as their concentrations alter. When comparing the preoperative state to the third postoperative day, the NLR rises (I-NLR & II-NLR *p* = 0.000463), although the NLR falls between the third and fifth days (II-NLR & III-NLR *p* = 0.007399), not to the preoperative level (I-NLR & III-NLR *p* = 0.009563).

#### 3.9.5. PLT

Between the preoperative period and the third postoperative day, as well as between the preoperative period and the fifth postoperative day, the platelet count (PLT) is decreased (I-PLT ([10^3^/µl] & II-PLT [10^3^/µl] *p* = 0.000132, I-PLT [10^3^/µl] & III-PLT [10^3^/µl] *p* = 0.000132). There were no significant changes between the third and fifth postoperative days (II-PLT [10^3^/µl] & III-PLT [10^3^/µl] *p* = 0.227331).

#### 3.9.6. MPV, P-LCR

There were no significant changes in MPV (mean platelet volume) across individual blood samples. When comparing P-LCR (platelet-large cell ratio) amongst samples, there was a significant difference between the preoperative period and the third postoperative day, where an increase in P-LCR between blood samples is seen. (I-P-LCR [%] & II-P-LCR [%] *p* = 0.007686).

#### 3.9.7. PCT

Between the pre-and post-operative periods, there was a significant decrease in PCT (platelet-crit) (I-PCT [%] & II-PCT [%] *p* = 0.000196, I-PCT [%] & III-PCT [%] *p* = 0.000151). The PCT level did not return to preoperative levels until the fifth surgical day (II-PCT [%] & III-PCT [%] *p* = 0.278708).

#### 3.9.8. PDW

The increase in PDW (platelet distribution width) between the preoperative period and the fifth postoperative day is the sole significant change in the PDW (I-PDW [fl] & III-PDW [fl] *p* = 0.013042). Other PDW relationships are not statistically significant.

#### 3.9.9. CRP

CRP levels increased significantly during the preoperative and postoperative periods (I-CRP [mg/L] & II-CRP [mg/L] *p* = 0.000140, I-CRP [mg/L] & III-CRP [mg/L] *p* = 0.000120). CRP did not decrease until the fifth postoperative day (II-CRP [mg/L] & III-CRP [mg/L] *p* = 0.765199).

#### 3.9.10. D-DIMER

The postoperative concentration of d-dimer is statistically higher than the preoperative value (I-D-DIMER [ng/ml] & II-D-DIMER [ng/ml] *p* = 0.002225, I-D-DIMER [ng/ml] & III-D-DIMER [ng/ml] *p* = 0.022232). The d-dimer concentration does not return to preoperative levels until the fifth postoperative day. No decrease in d-dimer concentration was observed between the 3rd and 5th postoperative day (II-D-DIMER [ng/ml] & III-D-DIMER [ng/ml] *p* = 0.463107).

#### 3.9.11. PROCALCITONIN

Procalcitonin levels increased significantly between the preoperative period and the third postoperative day (I-PROKALCITONIN [ng/ml] & II- PROKALCITONIN [ng/ml] *p* = 0.000293) and persist until the fifth postoperative day (I- PROKALCITONIN [ng/ml] & III- PROKALCITONIN [ng/ml] *p* = 0.000233). There was no significant in procalcitonin level difference between the third and fifth postoperative days (II- PROKALCITONIN [ng/ml] & III- PROKALCITONIN [ng/ml] *p* = 0.236631).

#### 3.9.12. PROTHROMBIN TIME, PROTHROMBIN INDEX

Prothrombin time was prolonged between the preoperative period and the third postoperative day (I-PROTHROMBIN TIME [s] & II- PROTHROMBIN TIME [s] *p* = 0.000536). Between the third postoperative day, the prothrombin time decreased to the preoperative level (I- PROTHROMBIN TIME [s] & III- PROTHROMBIN TIME [s] *p* = 0.081507, II- PROTHROMBIN TIME [s] & III- PROTHROMBIN TIME [s] *p* = 0.000438). The prothrombin index revealed a similar dependence. Between the preoperative period and the third postoperative day, the index increased (I-PROTHROMBIN INDEX [%] & II- PROTHROMBIN INDEX [%] *p* = 0.000536). The postoperative index decreased between the third and fifth reaching the preoperative level (I- PROTHROMBIN INDEX [%] & III- PROTHROMBIN INDEX [%] *p* = 0.062672, II- PROTHROMBIN INDEX [%] & III- PROTHROMBIN INDEX [%] *p* = 0.000438).

#### 3.9.13. INR

Between the preoperative period and the third postoperative day, the INR level is significantly prolonged (I-INR & II-INR *p* = 0.000342) Between the third and fifth postoperative days, there is a significant regression to the preoperative level of INR (II-INR & III-INR *p* = 0.000293). INR level normalizes on the 5th postoperative day (I-INR & III-INR *p* = 0.089421).

#### 3.9.14. FIBRINOGEN

The fibrinogen level does not fluctuate markedly, and only a significant elevation of fibrinogen is observed between the preoperative period and the fifth postoperative day (I- FIBRINOGEN [mg/dl] & III- FIBRINOGEN [mg/dl] *p* = 0.036386).

#### 3.9.15. APTT, APTT RATIO

Between the preoperative period and the third postoperative day, the APTT is significantly extended (I-APTT [s] & II-APTT [s] *p* = 0.030366). Between the third and fifth postoperative days, APTT decreases significantly (II-APTT [s] & III-APTT [s] *p* = 0.006211). On the fifth postoperative day, APTT measurements return to the preoperative level (I-APTT [s] & III-APTT [s] *p* = 0.747500). APTT ratio behaves similarly because it follows the same dependencies. the APTT ratio increases between the preoperative period and the third postoperative day (I-APTT RATIO & II-APTT RATIO *p* = 0.036386), and then statistically decreases between the third and fifth postoperative day (II-APTT RATIO & III-APTT RATIO *p* = 0.007449) reaching the preoperative level (I-APTT RATIO & III-APTT RATIO *p* = 0.732307).

## 4. Discussion

Due to neutrophils' major role as an inflammatory mediator, neutrophils are widely used as an indication of surgery stress or wound healing, as well as postoperative sepsis (19). It is well known that inflammatory processes occur after stent graft placement (20). This research is focused on neutrophil network activity during the pre-and postoperative period in relationship with selected inflammatory and coagulation parameters. Previous research on the impact of NETs included individuals with AAA (21). This is the first research that includes individuals with TAAA. In this study, we evaluate the neutrophil networks that are released in response to the inflammatory process that occurs in connection with the placement of a stent graft (20). All tested markers, such as dsDNA, ssDNA, and Cit-H3, were elevated in the postoperative period. We examine the ratio of NET markers to neutrophils. The intensity of NET production was observed to be independent of postoperative neutrophil expansion. Thus, in the postoperative phase, there is not only a considerable increase in neutrophils, but also a significant activation of these cells. A significant increase in NETs markers shortly after surgery indicates neutrophil activity and a postoperative NETosis. The only marker that returns to the pre-surgery level is Cit-H3. This marker is liberated to the bloodstream during the release of neutrophil nets and is the most specific mediator of this NETosis because it is not produced in any other mechanism (22). Extracellular DNA can be released into circulation not only in NETosis but also in the situation of disintegrating cells where the cell membrane is damaged. This process occurs in malignant illnesses, and cfDNA is even applied to predict patient survival (23). The remaining ssDNA and dsDNA marker levels do not return to normal until the fifth postoperative day when the last collection of blood was performed. Cit-H3 is a marker of inflammation, and the elevation indicates a reaction of the patient to stent graft implantation. Other studies have suggested that Cit-H3 histones are responsible for endothelial cell damage (24) that occurs during t-Branch placement within the aorta. The level of dsDNA and ssDNA does not return to normal after surgery, demonstrating the NETs ongoing remodeling of nets architecture. It could be speculated that the continuously high concentration of cfDNA on the fifth postoperative day, when the concentration of Cit-H3 has been normalized, might come from injured cells that participate in the development of a thrombus between the stent and the arterial wall. Thus, platelets, erythrocytes, and coagulation factors are attached to their NETs, NETs actively participate in the creation of thrombi. The process of thrombus remodeling between the endothelium and the implanted stent allows the aneurysm's diameter to be decreased over a longer time (25).

Unlike previous NETs studies, which examined cfDNA overall, we investigated separately single-stranded (ssDNA) and double-stranded DNA (dsDNA). So far, no research has been conducted on single-stranded DNA in the context of the NETs marker. SsDNA has significantly higher concentrations than dsDNA. We found that ssDNA level has a strong correlation in pre-and postoperative blood samples to dsDNA. Even though double-stranded DNA breaks are the most harmful and can be induced by radiation (26), this process occurs within the nucleus of the cell. In our study, we evaluated the concentration of DNA in the bloodstream, and the ss/dsDNA ratio did not vary between samples. However, it seems that radiation does not affect the disproportions of the level of ssDNA to dsDNA because the preoperative ratio is the same as the postoperative. We postulate that ssDNA could be a marker of NETs formation as similarly dsDNA. Further study is needed to determine if ssDNA is secreted with dsDNA during NETosis, or whether ssDNA results from breaking dsDNA after releasing dsDNA. In the study, NETosis growth in response to pathogen preoperative infection was excluded, due to the absence of clinical symptoms and negative preoperative pathogen testing (data not shown). In this study, NETosis markers were examined in the context of inflammatory processes. An increase in inflammatory markers subsequent to stent implantation could indicate the development of post-implantation syndrome (PIS). However, it is critical to distinguish when an increase in measured parameters is a pathology because the response to stent graft placement may vary from patient to patient. It is suggested that in addition to the absolute values used to diagnose PIS, such as fever (>38°C), procalcitonin (>0.5 ng/ml), CRP (>10 mg/L), and WBC (>12 000/ml), the long-term elevation of these parameters following surgery is more essential (27, 28). We found that, despite a significant increase in procalcitonin concentrations, its level is within the normal range which could be induced by surgery-related inflammation (29). Other parameters such as CRP, WBC, and neutrophils count were also elevated postoperatively, which could be indicated an enhancement in the inflammatory response after stent-graft placement. Significant elevation of CRP levels on the third and fifth postoperative days compared to the preoperative period suggest a significant inflammatory response in patients with TAAA in this study (30, 31). The number of neutrophils is significantly elevated in the postoperative phase compared to the level before surgery. The number of neutrophils does not correlate with any NETs parameters. A significant decline in lymphocytes is seen on the third and fifth postoperative days compared to the preoperative phase. Our study demonstrates NLR fluctuation with growth being significant on the third postoperative day and normalization beginning on the fifth postoperative day. Based on the NLR level it is seen that the highest inflammation response occurs on the third postoperative day. The decrease in lymphocytes is connected to an increase in cortisol level, which is seen after surgery, and in response to this immunosuppressive hormone, lymphocytes are redistributed to the lymphatic tissue (32). The NLR is gaining popularity in comparison to other criteria since it can indicate the increased risk of postoperative morbidity and poor outcomes following surgery (33). The main reason for its popularity is the simplicity of calculation because only two parameters are needed and each of which play important role in other mechanics. Lymphocytes represent immunosuppressive effects whereas neutrophils reflect an inflammatory response. The main downside of this parameter is the lack of clear cut-off points for this parameter (34). Neutrophil networks may contribute to the activation of the extrinsic pathway. NETs, due to their spatial configuration, create gaps to which such factors as fibronectin, fibrinogen, factor VWB, and factor XII can adhere. DNA and histones contained in the nets not only initiate the formation of fibrin but also alter its architecture, making it resistant to mechanical and enzymatic degradation. These networks provide a solid support for procoagulant factors and contribute to maintaining the stability of the thrombus (35). Numerous pre- and postoperative disturbances affect coagulation, fibrinolysis, and platelet activation as variables influencing hemostasis. The postoperative platelet count is lower than that in the preoperative state. Due to the prothrombotic environment which occurs in response to stent graft placement. It is worth mentioning that between platelets and neutrophils secreting NETs are many interactions. Platelets can stimulate NETs releasing through integrins, selectins, and chemokines, and active neutrophils encourage platelet activation resulting in a vicious circle. This prothrombotic condition promotes the development of a thrombus. Other research has indicated that antiplatelet treatments can stop the vicious spiral (36). The prothrombin time is used to analyze the extrinsic pathway, similar to INR which is also a reflection of this pathway. The initiation of double antiplatelet oral treatment on the third postoperative day resulted in the prolongation of prothrombin and INR. The APTT time after the surgery was prolonged, and the APTT ratio behaves similarly. The reason for the lengthening of the APTT is treatment with unfractionated heparin during and after surgery. APTT returns to the preoperative length on the fifth postoperative day.

After endovascular surgery, d-dimers, a recognized indicator of thrombosis, are dramatically raised (37). It is noteworthy that preoperative d-dimer concentrations are higher than in the healthy population. The dramatically higher level of d-dimer following surgery is caused by the release of d-dimer as a result of intravascular thrombus formation (38).

## 5. Limitations

The study's limitations include a small number of participants with TAAA who qualified for t-Branch placement due to final restrictions. Another limitation of this study is three-time points of blood collection; in order to more accurately describe the oscillation of the parameters, blood should be collected more frequently, e.g., every day. The last collection occurred on the fifth postoperative day, and the majority of the analyzed parameters did not return to their preoperative state; thus, in order to assess their oscillations, these markers should be evaluated at a later time.

## 6. Conclusion

NETs are secreted in response to the complex process which occurs after stent graft placement. All NETs markers are increased shortly after the surgery, and the histones are the first marker that returns to the preoperative phase. The lack of normalization of level dsDNA and ssDNA to the preoperational range until the fifth postoperative day demonstrates the reorganization of NETs. The increase. in postoperative NET formation was independent of neutrophil count elevation. The study investigates a novel NETosis marker which is ssDNA. In a patient with TAAA, the placement of a stent graft generates an inflammatory reaction in the appearance of an increase in inflammatory parameters. The activation of extracellular neutrophil traps is one of the characteristic markers of inflammation.

## Data Availability

The original contributions presented in the study are included in the article/Supplementary Material, further inquiries can be directed to the corresponding author.
